# Effect of Harvest Seasons on Biochemical Components and Volatile Compounds in White Teas from Two Cultivars

**DOI:** 10.3390/foods14101795

**Published:** 2025-05-18

**Authors:** Fan Huang, Haijun Wu, Fan Luo, Yingchun Wang, Yulong Ye, Yiyun Gong, Xianlin Ye

**Affiliations:** 1 Tea Research Institute, Sichuan Academy Agricultural Sciences, Chengdu 610066, China; huangfan@scsaas.cn (F.H.);; 2 Sichuan Academy Agricultural Sciences, Chengdu 610061, China

**Keywords:** white tea, biochemical components, volatile components, harvest seasons

## Abstract

The flavor profile of white tea emerges from the natural biochemical composition of its tender leaves, a delicate balance profoundly shaped by seasonal growing conditions and tea cultivars. However, the effects of harvest seasons on biochemical and volatile compounds in white teas in southwestern China have not been fully analyzed at present. This study investigated the sensory characteristics, biochemical components, and volatile compounds of ‘Sanhua1951’ spring white tea (SH-S), ‘Sanhua1951’ autumn white tea (SH-A), ‘Fudingdabai’ spring white tea (FD-S), and ‘Fudingdabai’ autumn white tea (FD-A). The results showed that the sensory quality (appearance, taste, and aroma) scores of spring tea were higher than those of autumn tea. Spring teas exhibited significantly higher epigallocatechin, soluble sugar, and amino acid levels than autumn teas (*p* < 0.05), whereas autumn teas contained greater contents of epicatechin gallate, catechin, caffeine, and polyphenols (*p* < 0.05), which were responsible for the differences in taste quality observed between samples with different harvest seasons. A total of 90 volatile compounds in four groups were identified through HS-SPME–GC–MS analysis, and spring white teas contained higher contents of and variability in volatile compounds than autumn white teas. According to the OPLS-DA model, 52 and 57 differential volatile compounds (VIP > 1, *p* < 0.05, and fold change ≥ 2 or ≤0.5) were identified in SH-S vs. SH-A and FD-S vs. FD-A, including (*Z*)-linalool oxide, (*E*)-linalool oxide, styrene, phenylethyl alcohol, (*Z*)-citral, etc. The odor active value (OAV) results indicated that 30 key differential volatile compounds (OAV > 1) were determined in four groups, among which β-ionone, 5,6-epoxy-β-ionone, linalool, and (*E*)-linalool oxide exhibited particularly high OAVs and contributed more pekoe aroma and floral sensory characteristics. Notably, (*E*)-linalool oxide, (*Z*)-jasmone, and δ-cadinene were identified in each cultivar. These findings suggest their potential as seasonal markers, paving the way for the development of white tea ’Sanhua1951’ and ’Fudingdabai’.

## 1. Introduction

Tea (*Camellia Sinensis*, L.) is one of the most popular drinks worldwide and can be grouped into black, green, yellow, dark, white, and oolong teas based on processing methods, sensory qualities, and raw materials [1]. White tea, a traditional Chinese tea derived from Fujian Province, has witnessed a rapid increase in consumer preference and production expansion in recent years since its revealed health functions [2,3,4]. By 2023, the production of white tea in China reached 10^6^ tons, accounting for more than 3% of the total tea yield [5]. Due to the quality of white tea requiring large buds, more trichomes, mellow taste, and fresh aroma, there are certain requirements for the selection of raw materials of the tea cultivars [6]. In addition to traditional tea cultivars suitable for white tea, such as ‘Fudingdabai’ (*Camellia sinensis* L. Kuntze), fresh tea leaves from more cultivars were made into white tea in a number of provinces in China [7,8,9]. As a major tea province located in southwestern China, Sichuan Province also utilizes the multiple tea cultivars in this area to manufacture white tea. ‘Sanhua1951’ is a new *Camellia sinensis* cultivar (approval number: 2015001), which was systematically bred from the individual plants found in the tea garden of Sichuan small- and medium-sized leaf groups in Chengjia Town, Sichuan Province. It has been widely planted in Chengdu, Ya ‘an, Bazhong, and other regions. Its characteristics such as more trichomes in its leaves, early germination, long picking period, vigorous growth, and strong insect resistance make ‘Sanhua1951’ an excellent candidate for white tea production. Currently, in southwestern China, fresh tea leaves utilized for white tea processing are predominantly sourced from spring- and autumn-harvested ‘Sanhua1951’ and ‘Fudingdabai’.

In addition to the tea cultivar, the influence of seasons on the chemical composition of tea has also been reported in six kinds of teas [10,11,12]. The environmental conditions such as temperature, light, and humidity in different seasons affect the tea quality at the pre-harvested stage, which includes physiology [13], composition, and the content of non-volatile and volatile substances in fresh tea leaves [14,15]. Spring green tea is usually considered to be better than summer green tea [16]. For example, steamed green teas harvested in spring contain a higher abundance of volatile compounds with ‘floral/green/herbal/fruity/fresh/sweet’ aromas than summer and autumn tea [14]. Yinghong No. 9, a renowned large-leaf black tea from South China, exhibits superior taste and color quality in its summer variety compared to spring tea, with the latter showing lower catechin content but higher levels of caffeine, total amino acids, theabrownin, and, notably, glutamic acid content [17]. A study reported that fresh tea leaves in spring contain more highly unsaturated fatty acids, phospholipids, flavor-enhancing amino acids, phenolic acids, theanine, catechins, gallotannins, and aroma precursors encompass key fragrant components, which may give spring-harvested tea a better flavor for processing into black tea [18]. Zhou et al. examined 266 ‘Tieguanyin’ oolong tea products harvested in both spring and autumn and found that over half of the identified key seasonal discriminant metabolites happened to be crucial for determining the sensory grade [19]. Studies on white teas from different seasons have found that there are differences in chemical compositions, aroma components, in vitro antioxidant capacity, and sensory quality [4,20]. However, white tea produced using ‘Fudingdabai’ in southwestern China has not been fully investigated, and its dominant flavor ingredients remain uncovered. To date, little research has been reported comparing the biochemical components and volatile components of ‘Sanhua1951’ white tea harvested in different seasons.

In this study, the fresh leaves of ‘Sanhua1951’ and ‘Fudingdabai’ in spring and autumn were made into white teas (SH-S, SH-A, FD-S, FD-A). Through sensory evaluation, biochemical component analysis, volatile compound determination, and odor activity value (OAV) analysis, the effect of harvest seasons on the quality of white tea was examined. The results of this study offer comprehensive insights into the flavor quality in white tea from different harvest seasons and cultivars, thereby establishing a theoretical foundation for directional processing technology.

## 2. Materials and Methods

### 2.1. Chemical Reagents

The deionized water used for experiments was prepared by a Milli-Q Water Purification System (Millipore, Billerica, MA, USA). Folinphenol, ninhydrin, anthrone, and methanol (AR grade) were purchased from China national pharmaceutical group, Shanghai Chemical Reagent Co., Ltd. (Shanghai, China). The standards epicatechin (EC), epigallocatechin (EGC), catechin (C), gallocatechin (GC), epicatechin gallate (ECG), epigallocatechin gallate (EGCG), catechin gallate (CG), and gallocatechin gallate (GCG) (purity ≥ 95%) were supplied by Sigma-Aldrich (St. Louis, MO, USA). HPLC-grade acetic acid, methanol, and acetonitrile were obtained from Aladdin Biological (Shanghai, China).

### 2.2. Tea Sample Preparation

*Camellia sinensis* cv. ‘Sanhua1951’ and ‘FudingDabai’ tea plants were cultivated in Pingle Town, Qionglai, Sichuan Province, where they had been growing for 4 years in a single plantation (30.41º N, 103.30º E). Raw materials of fresh tea leaves (single-bud only) of two cultivars were harvested in March 2023 (sunny, 28–30 °C) and manufactured into white teas using the same processing steps. The manufacturing steps were as follows: approximately 5 kg of fresh tea leaves were evenly spread on a customized withering tank (length: 10 m and width: 1 m) with a leaf spreading thickness of approximately 2 cm; they were withered indoors for at least 36 h (air temperature: 24–27 °C, air humidity: 60–70%); the tea leaves were dried at 75 °C for 90 min using aroma-enhancing equipment (JY-6CHZ-7B Jiayou Machinery Co., Ltd., Fujian, China). The same practices were repeated in August 2023 (sunny, 33–35 °C). The four groups of tea were named SH-S (‘Sanhua1951’, spring), FD-S (‘Fudingdabai’, spring), SH-A (‘Sanhua1951’, autumn), and FD-A (‘Fudingdabai’, autumn), which had 3 biological replicates each.

### 2.3. Sensory Evaluation

The sensory assessments were conducted independently by eight certified tea assessors with 5–20 years of professional experience in tea science, according to the white tea review method in Chinese National Standard GB/T 23776-2018 ‘Tea Sensory Review Methods’ [21]. Three grams of tea were weighed, using a tea–water ratio of 1:50. The tea was placed in the designated teacup, filled with boiling water, covered, and brewed for 5 min. Subsequently, the tea broth was filtered at a consistent speed, and sensory evaluations of comments and scores were executed based on leaf appearance, aroma, and taste.

### 2.4. Detection of Biochemical Components

The determination of total polyphenol and catechin content was performed under the Chinese National Standard GB/T 8313-2013 [21]. The determination of caffeine was performed under the Chinese National Standard GB 5009.139-2014 [21]. The determination of amino acid content was performed following the Chinese National Standard GB/T 8314-2013 [21]. The determination of soluble sugar content was performed using anthrone colorimetry [20].The determination of water extract content was performed following the Chinese National Standard GB/T 8305-2013 [21].

### 2.5. Extraction and Determination of Volatile Compounds

Volatile compounds were extracted via a method referred to in a previous study with slight adjustments [20]. Tea powders (5.0 g) were first placed in a 100 mL glass by adding 10 μL ethyl caprate (100 mg/L) as an internal standard. After adding 50 mL boiling deionized water, the vial was immediately sealed with a cover and equilibrated for 5.0 min at 60 °C. Next, a DVB/CAR/PDM coating fiber (50/30 μm; 1 cm; Supelco, Inc, Bellefonte, PA, USA) was inserted into the vial to adsorb the volatile compounds for 50 min. Finally, the volatile compounds were desorbed at 240 °C for 3 min for GC–MS analysis. The GC–MS analysis was carried out using a GC system (7890A, Agilent Technologies, Santa Clara, CA, USA) coupled with an MS detector (5975C, Agilent Technologies, Santa Clara, CA, USA). The column was an HP-5MS type (30 m × 250 μm × 0.25 μm, Agilent Technologies; Santa Clara, CA, USA), and the carrier gas was helium (99.999%) with a constant flow of 1.0 mL/min. The GC temperature-rise procedures were as follows: 50 °C held for 5 min, slowly increased to 180 °C at 3 °C/min, and then increased to 250 °C at 10 °C/min and held for 3 min. The splitless injection mode was applied. The MS conditions were as follows: the ionization mode was EI; the ion source temperature was 230 °C; the quadrupole temperature was 150 °C; the mass scan scope was 50–550 m/z; the electron impact ionization was −70 eV. The concentrations of volatiles were calculated using the following formula: *Ce* (μg/L) = (*PA*/*PAIS*) × 10 μL × 100 mg/L, where *Ce*, *PA*, and *PAIS* represent the concentrations of the examined compound, peak area of volatile, and peak area of IS, respectively. The limit of detection (LOD) was 0.3 × 10^−11^ g/L, and the limit of quantification (LOQ) was 1 × 10^−11^ g/L.

### 2.6. Calculation of Odor Activity Values (OAVs)

The OAVs of volatile compounds were calculated according to the formula OAV = C/OT, in which C is the concentration of a volatile compound in tea and OT is its odor threshold in water from the literature. Volatile compounds with an OAV > 1 are generally considered to have a contribution to the aroma characteristics of tea [22].

### 2.7. Statistical Analysis

The figures, histograms, and ring charts were generated with the Origin software 2024. Principal component analysis (PCA) using Pareto mode and orthogonal projections to latent structures–discriminant analysis (OPLS-DA) using UV mode were performed using Simca-P 13.0 software (Umetrics AB, Umea, Sweden). Variable importance for projection (VIP) value was calculated using OPLS-DA. The Venn diagram and the significant differences were calculated by a two-factor analysis of variance (ANOVA), and volcano plots were obtained using the Metware Cloud online platform (https://cloud.metware.cn, accessed on 28 March and 1 April 2025).

## 3. Results and Discussion

### 3.1. Tea Sample Sensory Evaluation

The sensory quality of ‘Sanhua1951’ and ‘Fudingdabai’ harvested in spring and autumn was evaluated using traditional sensory evaluations, as depicted in Table 1. For ‘Sanhua1951’ cultivars, SH-S tea samples displayed a more robust appearance with umami and a slightly sweet taste. Also, the fresh odor, bitterness, and astringency are more obvious than SH-A tea samples. The scores of the appearance, taste, and aroma of SH-S were higher than SH-A (*p* < 0.05). For ‘Fudingdabai’ cultivars, the scores of the appearance, taste, and aroma of FD-S were higher than FD-A (*p* < 0.05). Thus, this study found that spring tea was superior to autumn tea. Comparing ‘Sanhua1951’ to ‘Fudingdabai’, the appearance scores of SH-S and SH-A were higher than FD-S and FD-A (*p* < 0.05), while the taste score and aroma score were lower (*p* < 0.05). No significant difference in aroma scores were observed between SH-S and FD-S or between SH-A and FD-A (*p* < 0.05), nor were significant differences detected in taste scores between SH-A and FD-A (*p* < 0.05). Therefore, the sensory evaluations of white teas were mainly due to seasonal factors rather than the two tea cultivars.

### 3.2. Analysis of Main Biochemical Components

The major biochemical components such as catechins, caffeine, tea polyphenols, and amino acids in teas are greatly affected by harvest seasons and tea cultivars [23]. As shown in Table 2, comparing SH-S to SH-A, the contents of EC, ECG, polyphenols, and caffeine in SH-S were significantly lower (*p* < 0.05) than SH-A, and the contents of EGC, water extracts, soluble sugar and free amino acids in SH-S were significantly higher (*p* < 0.05) than SH-A. However, there was no significant difference in EGCG between SH-S and SH-A. Comparing FD-S to FD-A, the contents of EC, ECG, caffeine, and polyphenols in FD-S were significantly lower (*p* < 0.05) than FD-A, and the contents of EGC, EGCG, free amino acids, and soluble sugar in FD-S were significantly higher (*p* < 0.05) than FD-A. Conversely, there was no significant difference in water extract contents between FD-S and FD-A (Table 2). The biochemical components of the two cultivars exhibited consistent trends in spring and autumn tea, with significant differences observed solely in the levels of EGCG and water extract contents. The results of this study were consistent with the seasonal variation trend in Fujian white tea [4,24]. According to previous studies, the biochemical components of teas are greatly affected by harvest seasons [25,26,27]. Light intensity and temperature can synergistically regulate catechin synthesis [28]. The temperature in spring and autumn is not much different, and it is guessed that the light intensity is the main reason for the change in components. The observed biochemical differences in compositional disparities between spring and autumn tea samples in this study, processed under comparable temperature conditions, are hypothesized to result primarily from differential light intensity exposure during cultivation.

It was worth noting that ECG content in SH-A was 36 times higher than that of SH-S, and that ECG content in FD-A was 68 times higher than that of FD-S in this study. ECG is the key component in tea soup, which has the lowest bitterness detection threshold and the most intense taste among catechins [29], and the content was affected by factors such as tea cultivars, growing environment, processing, and so on [30,31]. In addition, catechins, major polyphenols in tea, are known for contributing to the astringent flavor of the tea broth [32]. Therefore, spring teas (SH-S, FD-S) were less astringent than autumn teas (SH-A, FD-A). The presence of astringency was also confirmed through a sensory evaluation of the SH-A sample (Table 1), which is consistent with the observed chemical profile. Free amino acids, especially theanine, are responsible for the tea’s umami taste, and the taste intensity increases with the amino acid concentration [33]. In this study, spring teas (SH-S, FD-S) had higher (*p* < 0.05) amino acid and lower (*p* < 0.05) polyphenol contents than autumn teas (SH-A, FD-A) (Table 2).

### 3.3. Overall Determination of Volatile Compounds

**Figure 1:**
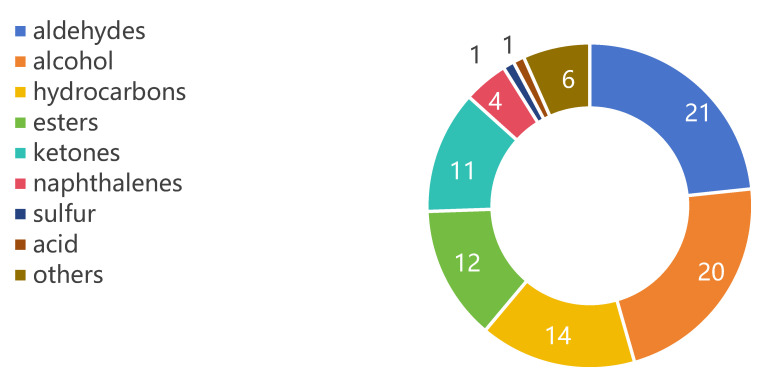
Numbers of volatile components of different categories in four groups.

The tea aroma is a specific fragrance of tea formed by the combination of different aromatic substances in different concentrations on the olfactory nerve. The volatile compounds were detected and analyzed by headspace solid-phase micro-extraction–gas chromatography–mass spectrometry (HS-SPME–GC–MS) to differentiate the differences in volatile compounds among white teas. A total of 90 volatile components were identified (Table S1), classified into 9 categories (Figure 1), including sulfur, aldehydes, alcohols, ketones, acid, hydrocarbons, esters, naphthalenes, and others. The quantities of individual categories are illustrated in Figure 1.

**Figure 2:**
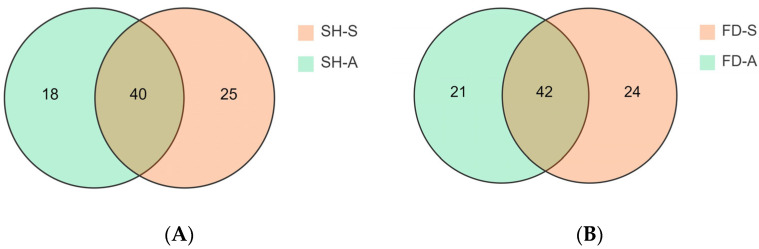
Venn diagrams of four tea samples. (**A**) The number distribution of volatile compounds in ‘Sanhua1951’ between spring and autumn; (**B**) The number distribution of volatile compounds in ‘Fudingdabai’ between spring and autumn.

There were 65, 66, 58, and 63 volatile compounds identified from SH-S, FD-S, SH-A and FD-A, respectively. As shown in Table S2, the numbers of aldehydes (19, 19, 18, 18) in four groups (SH-S, FD-S, SH-A, FD-A) were highest among the categories, and the numbers of alcohols (11, 13, 15, 16) were second in four groups (SH-S, FD-S, SH-A, FD-A). The distribution of volatile compounds in two tea cultivars in different harvest seasons was visualized using a Venn diagram (Figure 2). Clearly, there are a total of 40 common volatile compounds between SH-S and SH-A and a total of 42 common volatile compounds between FD-S and FD-A.

**Figure 3:**
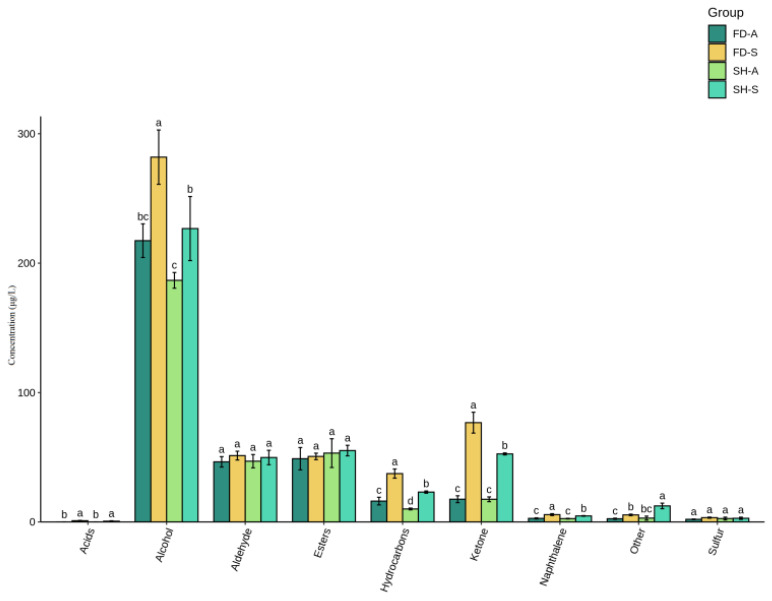
The concentration of volatile compounds of different categories from white tea samples. SH-S: Sanhua1951, spring; FD-S: Fudingdabai, spring; SH-A: Sanhua1951, autumn; FD-A: Fudingdabai, autumn. Different lowercase letters in the same line indicate a significant difference among white teas (*p* < 0.05).

As depicted in Figure 3, the analysis revealed a descending order of total volatile compounds across the samples. Specifically, FD-S exhibited the highest total volatile content with 513.440 ± 20.693 μg/L (Table S1). This was followed by SH-S, with a total volatile content of 428.001 ± 35.854 μg/L (Table S1). FD-A and SH-A showed relatively low values of 352.982 ± 23.034 μg/L and 321.278 ± 23.722 μg/L (Table S1), respectively. Alcohols had the highest content in the total volatile components in the order of FD-S (277.970 ± 17.246 μg/L) > SH-S (222.436 ± 20.121 μg/L) > FD-A (218.201 ± 11.173 μg/L) > SH-A (186.700 ± 4.954 μg/L). Moreover, among the content of alcohols, linalool was the highest (76.755–102.197 μg/L), followed by geraniol (52.084–108.070 μg/L), and the above substances all showed floral aroma (Table S1). The content of ketones varied greatly in the four groups of samples, followed by FD-S (76.789 ± 6.59 μg/L) > SH-S (52.613 ± 0.653 μg/L) > SH-A (17.539 ± 1.507 μg/L), FD-A (17.591 ± 2.182 μg/L). There were significant differences in contents of hydrocarbons between each other (*p* < 0.05). Meanwhile, there were no significant differences in contents of aldehyde, esters, and sulfur for each group (*p* < 0.05).

To ‘Sanhua1951’ white teas, the contents of acids, alcohol, hydrocarbons, ketone, naphthalene, and sulfur in SH-S were significantly higher (*p* < 0.05) than in SH-A. To ‘Fudingdabai’ white teas, the contents of acids, hydrocarbons, ketone, naphthalene, and others in FD-S were significantly higher (*p* < 0.05) than in FD-A (Figure 3). In conclusion, autumn teas had lower contents of aldehyde, alcohol, ketone, and total volatile components. Autumn-harvested teas exhibited higher ester content than spring-harvested ones. This is attributed to esters mainly originating from the lipoxygenase pathway and amino acid metabolism, processes that typically intensify at maturity [34]. It was noteworthy that acids were not detected in autumn teas (Figure 3). This also explained why the flavor score of autumn teas was lower than that of spring teas in the sensory evaluation results (Table 1). The results of this study were consistent with previous studies, where the tea’s volatile components were greatly affected by harvest seasons and tea cultivars [35,36]. The comparative analysis of white teas produced from two cultivars revealed that ‘Sanhua1951’ teas consistently exhibited a higher content of volatile compounds than ‘Fudingdabai’ across both spring and autumn seasons. In this study, the volatile components of white teas were mainly due to seasonal factors rather than the two tea cultivars.

**Figure 4:**
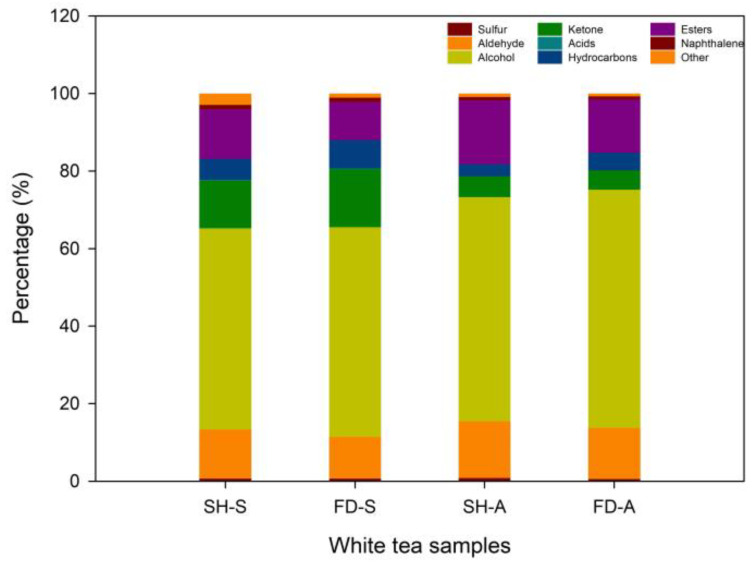
Percentage of volatile components of different categories from white tea samples. SH-S: Sanhua1951, spring; FD-S: Fudingdabai, spring; SH-A: Sanhua1951, autumn; FD-A: Fudingdabai, autumn.

As shown in Figure 4, alcohols (51.971%, 54.137%, 57.859%, 61.533%), aldehydes (12.644%, 10.750%, 14.559%, 13.110%), ketones (12.293%, 14.955%, 5.435%, 4.961%), and esters (12.896%, 9.868%, 16.496%, 13.79%) were identified as the predominant volatile compounds across the four groups (SH-S, FD-S, SH-A, FD-A). This composition is consistent with findings from prior white tea studies. In particular, similar to Dianhong tea, the proportion of alcohol in the four groups was the highest among the categories, which may be related to the genetic basis of tea cultivars and the growing environment [37]. It is worth noting that, as shown in Figure 4, the proportions of alcohols were higher in the autumn teas (SH-A, FD-A) than in the spring teas (SH-S, FD-S), while the total contents of these volatile compounds were lower in the autumn teas than in the spring teas (Figure 3). This differences between quantitative and qualitative value of alcohols correspond with different value of ketone but in opposite directions. The proportions of ketones were higher in the spring teas then in the autumn teas. Obviously, significant differences were observed in the proportions of volatile compounds between the spring and autumn harvests of white teas from the two cultivars.

### 3.4. PCA and OPLS-DA Statistical Analyses of the Volatile Compounds

**Figure 5:**
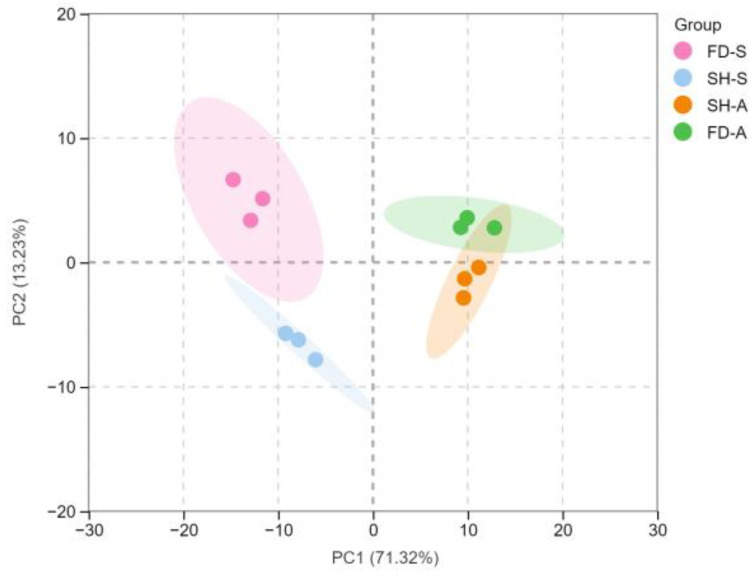
Principal component analysis (PCA) score plot for volatile compounds of the four white teas. SH-S: Sanhua1951, spring; FD-S: Fudingdabai, spring; SH-A: Sanhua1951, autumn; FD-A: Fudingdabai, autumn.

PCA is the principle of data dimension reduction, which mainly studies the material, its corresponding variables, and the potential relationship with each cultivar [38]. PCA was performed on the 90 volatile components detected in the samples of white tea, and PCA was performed separately. The results (Figure 5) showed that four groups (SH-S, FD-S, SH-A, FD-A) can be clearly divided into four regions. Meanwhile, the samples in the two seasons are far apart, indicating that there is a big difference in the aroma composition of white teas in spring and summer. It can be seen from Figure 5 that the variance contribution rates of PC1 and PC2 are 71.3% and 13.2%, respectively, and the cumulative contribution rate reaches 84.5%. Notably, PC1 can better represent the information reflected by the original data.

**Figure 6:**
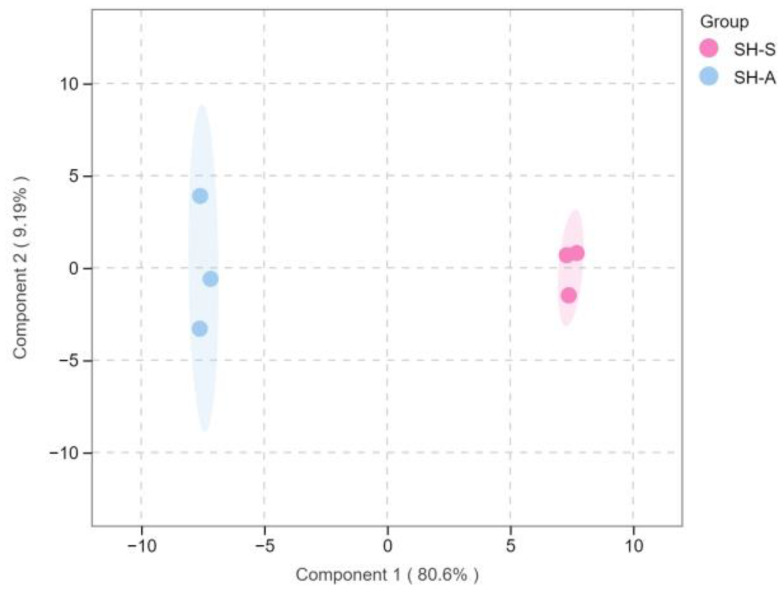
The orthogonal projections to latent structures–discriminant analysis (OPLS-DA) score plot for SH-S vs. SH-A. SH-S: Sanhua1951, spring; SH-A: Sanhua1951, autumn.

**Figure 7:**
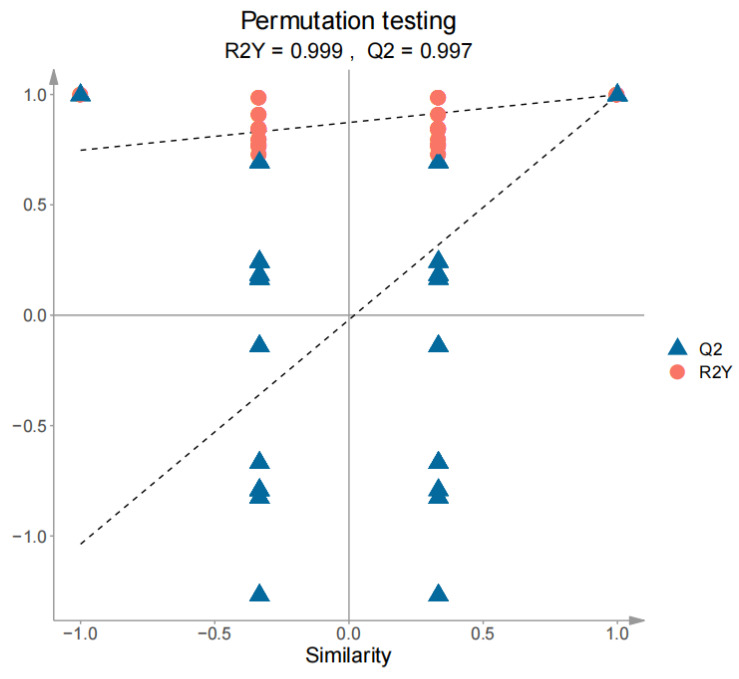
Cross-validation model for SH-S vs. SH-A. SH-S: Sanhua1951, spring; SH-A: Sanhua1951, autumn.

In order to further explore the different volatile components of white teas in ‘Sanhua1951’ and ‘Fudingdabai’, a supervised pattern recognition method, orthogonal projections to latent structures–discriminant analysis (OPLS-DA), was introduced. The OPLS-DA score plot (R^2^X = 0.806, R^2^Y = 0.998, Q^2^ = 0.993) demonstrated that SH-S and SH-A were clearly distinguished in Figure 6, which was consistent with the PCA results. Subsequently, the accuracy of the model was tested with a 200-permutation test (R^2^ = 0.873, Q^2^ = −0.020), and there was no overfitting of the model (Figure 7).

**Figure 8:**
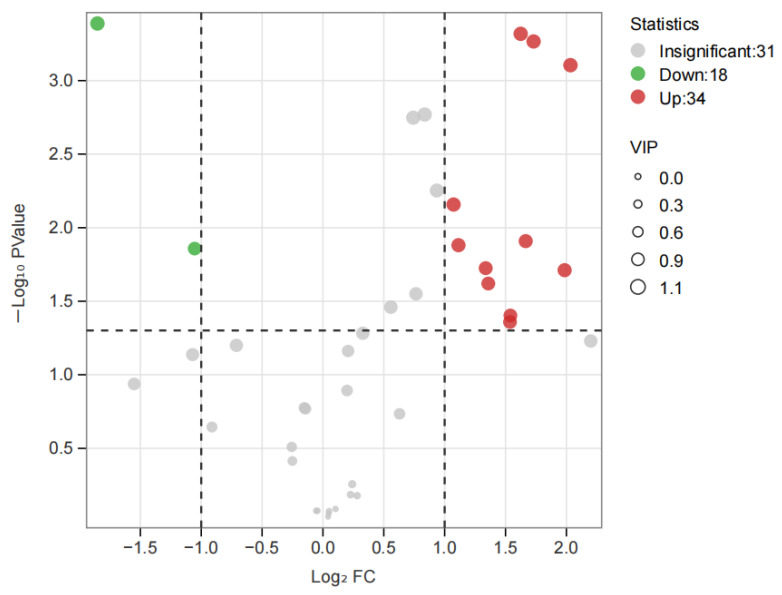
Volcano plot of SH-S vs. SH-A. SH-S: Sanhua1951, spring; SH-A: Sanhua1951, autumn.

**Figure 9:**
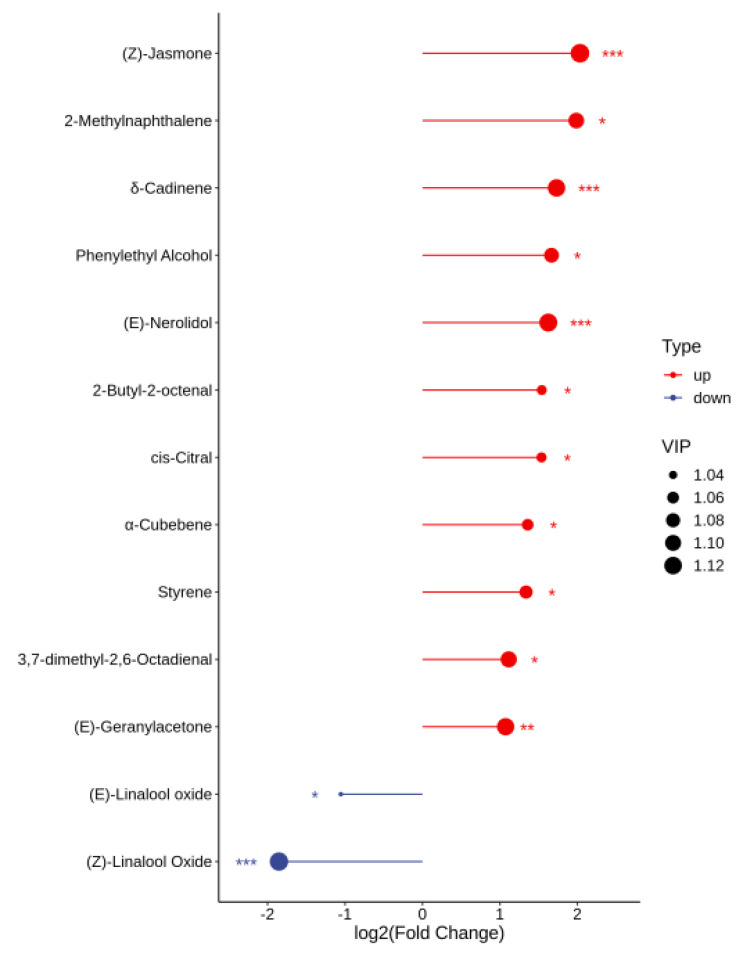
Score plot of key volatile compounds in SH-S vs. SH-A. SH-S: Sanhua1951, spring; SH-A: Sanhua1951, autumn. Add asterisks (*) on the graph according to the size of the *p* value, *p* < 0.05 = “*”, *p* < 0.01 = “**”, *p* < 0.001 = “***”.

The variables important in projection (VIP) were further utilized to focus on the volatile fraction explanation of key aroma compounds in white tea samples from two seasons using VIP > 1 as a criterion, where the larger VIP indicated that the key aroma compounds contributed more to the differentiation of white tea samples. As shown in Table S3, there were a total of 60 compounds with VIP > 1, which were the best discriminators between SH-S and SH-A, including the characteristic components of different tea cultivars. To investigate the impact of harvest seasons on white teas, significantly differential volatile components were selected according to the principle of *p* < 0.05, VIP > 1, and fold change ≥2 (up-regulated) or ≤ 0.5 (down-regulated) in each cultivar (Table S4). For ’Sanhua1951’, there were 52 differential volatile compounds in SH-S vs. SH-A (34 upregulated and 18 downregulated) (Figure 8). As shown in Figure 2A, there were 18 volatile compounds detected only in SH-S and 25 volatile compounds detected only in SH-A. In addition to the above-mentioned compounds, the comparison of other significantly differential volatile compounds was performed (Figure 9), including (*Z*)-linalool oxide, (*E*)-linalool oxide, styrene, phenylethyl alcohol, (*Z*)-citral, 3,7-dimethyl-2,6-octadienal, 2-methylnaphthalene, ɑ-cubebene, 2-butyl-2-octenal, (*Z*)-jasmone, (*E*)-geranylacetone, *δ*-cadinene, and (*E*)-nerolidol.

**Figure 10:**
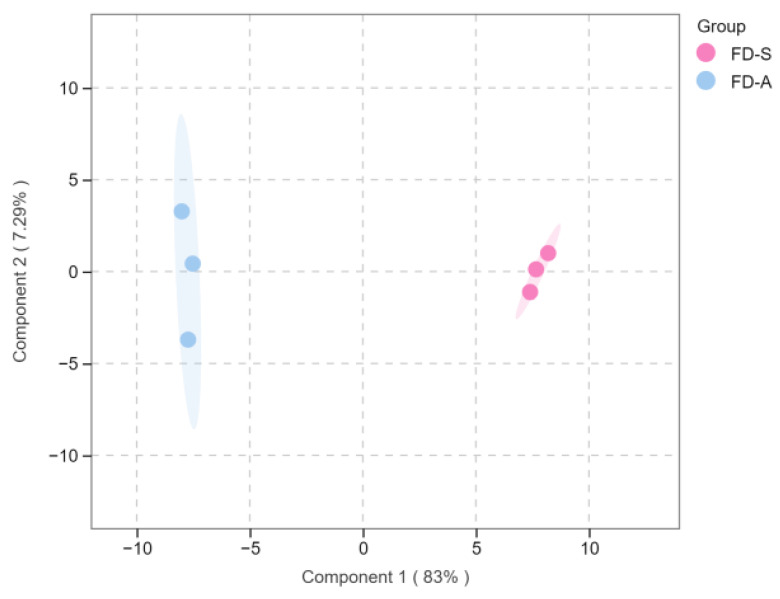
The orthogonal projections to latent structures–discriminant analysis (OPLS-DA) score plot for FD-S vs. FD-A. FD-S: Fudingdabai, spring; FD-A: Fudingdabai, autumn.

**Figure 11:**
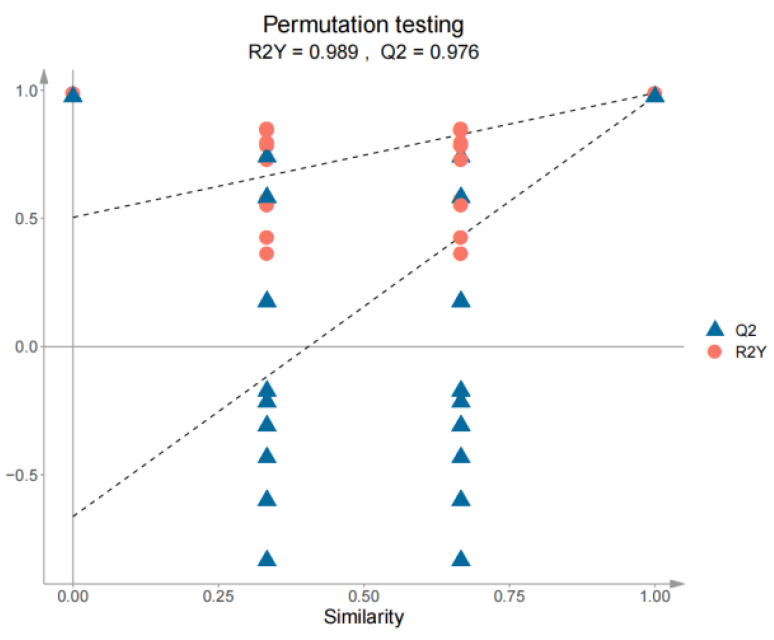
Cross-validation model of FD-S vs. FD-A. FD-S: Fudingdabai, spring; FD-A: Fudingdabai, autumn.

As shown in Figure 10, OPLS-DA (R^2^X = 0.830, R^2^Y = 0.994, Q^2^ = 0.987) was performed to analyze the significantly different volatile compounds between FD-S and FD-A. The permutation test (n = 200) revealed intercepts of R^2^ at 0.503 and Q^2^ at −0.663, and there was no overfitting for the model (Figure 11).

**Figure 12:**
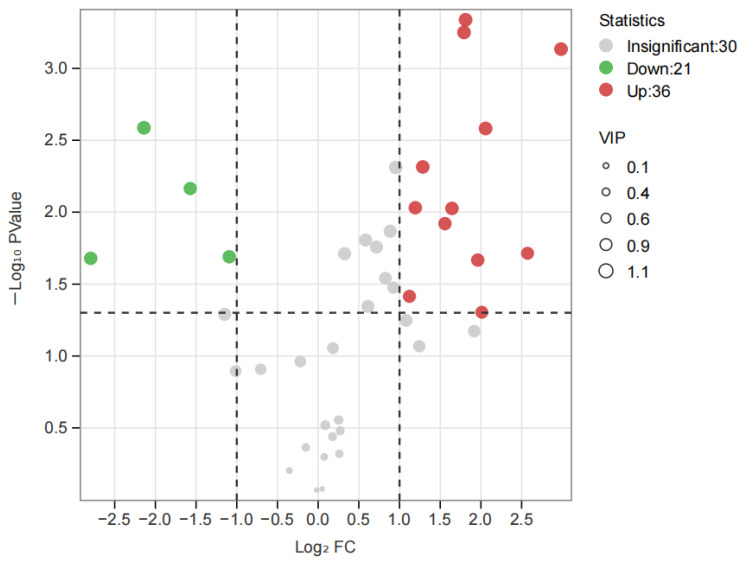
Volcano plot in FD-S vs. FD-A. FD-S: Fudingdabai, spring; FD-A: Fudingdabai, autumn.

**Figure 13:**
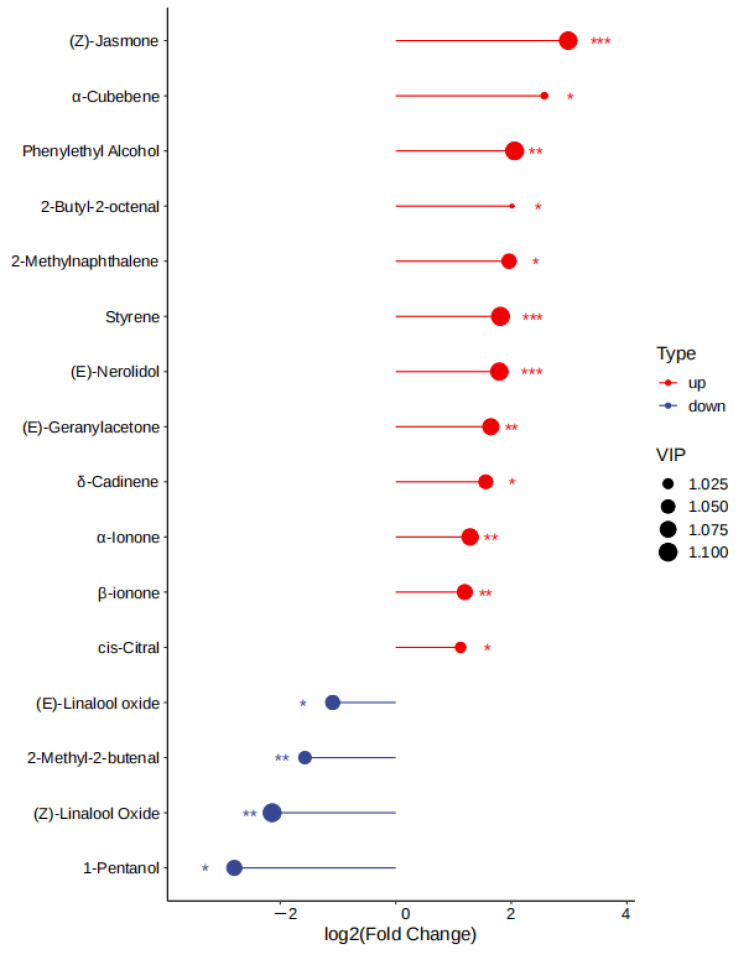
Score plot of key volatile compounds in FD-S vs. FD-A. FD-S: Fudingdabai, spring; FD-A: Fudingdabai, autumn. Add asterisks (*) on the graph according to the size of the *p* value, *p* < 0.05 = “*”, *p* < 0.01 = “**”, *p* < 0.001 = “***”.

As shown in Table S5, there were a total of 65 compounds with VIP > 1, which were the best discriminators between FD-S and FD-A, including the characteristic compounds of different tea cultivars. As shown in Figure 12, for ‘Fudingdabai’, based on the principle of *p* < 0.05, VIP > 1 and fold change ≥ 2 (up-regulated) or ≤ 0.5 (down-regulated), 57 differential volatile components in FD-S vs. FD-A (36 upregulated and 21 downregulated) (Table S6). As shown in Figure 2B, there were 24 volatile compounds detected only in FD-S, and 21 volatile compounds detected only in FD-A. In addition to the compounds mentioned above, a comparative analysis of other significantly differential volatile compounds was conducted (Figure 13), including 2-methyl-2-butenal, 1-pentanol, (*Z*)-linalool oxide, (*E*)-linalool oxide, styrene, phenylethyl alcohol, cis-citral, 2-methylnaphthalene, α-cubebene, 2-butyl-2-octenal, (*Z*)-jasmone, α-ionone, (*E*)-geranylacetone, β-ionone, δ-cadinene, and (*E*)-nerolidol. Moreover, these were the main sources of these aforementioned compound changes. Volatile terpenoids contributed to floral fragrance, primarily originating from the methylerythritol phosphate pathway, and their content in tea varies significantly across different producing regions [39]; examples include linalool oxide, α-cubebene, δ-cadinene, and (*E*)-nerolidol. The carotenoids in fresh tea leaves undergo nonenzymatic degradation (for instance, pyrolysis, isomerization, aromatization, cyclazation, and polymerization) and enzymatic oxidation to produce abundant aromatic compounds under different growth environments and harvest seasons, such as (*E*)-geranylacetone and carotenoid-derived aroma substances. Phenylethyl alcohol is an important volatile compound in white tea [40], primarily released through the hydrolysis of glycosides or the shikimic acid synthesis pathway. The contents of phenylethyl alcohol were lower in autumn teas than spring teas (Figure 13). (*Z*)-jasmone, a compound with floral notes, is the product of α-linolenic acid. Through distinct metabolic pathways involving enzymes such as alcohol dehydrogenase, allene oxide synthase, and lipoxygenase, these biochemical processes collectively lead to the formation of key volatile aroma compounds [41]. The contents of (*Z*)-jasmone were lower in autumn teas than spring teas (Figure 13). Based on the relative abundance of these aroma-related compounds, their odor characteristics, and prior studies [7,8,9], the change in the levels of the above-mentioned compounds play a pivotal role in shaping the aroma profile of white teas harvested in spring versus autumn. These compounds are likely responsible for the distinct ‘floral/herbal/fruity/fresh’ aromatic characteristics observed in these teas.

### 3.5. OAV Analysis of the Differential Aroma Substances

Numerous volatiles have been found in various foods, and only a limited number of volatiles are considered key aroma compounds that significantly influence the overall aroma profile [42]. The quantitative abundance and threshold of aroma-related compounds in tea soup have a great contribution to the quality characteristics and flavor of tea [43]. OAVs were used to assess the contribution of aroma compounds to the aroma of tea samples, and aroma compounds with an OAV > 1 were considered to be key flavor substances [44]. In total, there were 30 out of 90 volatile compounds with OAV > 1, 19 of which were commonly shared volatile compounds among the four groups. As shown in Table 3, there were 26, 22, 25, and 23 volatile compounds with OAV > 1 in SH-S, SH-A, FD-S, and FD-A, respectively.

In the 30 above-mentioned volatile compounds, most of them had significant differences (*p* < 0.05) among the four groups (SH-S, FD-S, SH-A, FD-A) in their contents (Table 3). Meanwhile, there were no significant differences (*p* < 0.05) among the four samples (SH-S, FD-S, SH-A, and FD-A) in the contents of eight volatile compounds with OAV > 1, including hexanal, nonanal, (*E*)-2-nonenal, decanal, oct-1-en-3-ol, ocimene, methyl salicylate, and ethyl nonylate, contributing significantly to the formation of the ‘aged/oily/herbal’ fragrance characteristics of white tea [45].

Among the 30 volatile compounds in the four groups, the top volatile, with OAV > 1, was β-ionone, with a numerical range of 3375.147–1439.131 (Table 3). β-ionone has an obvious floral aroma and plays a decisive role in the formation of the good flavor of various white teas [46]. In this study, the OAV of β-ionone in SH-S was significantly higher than that in SH-A, while the OAV of β-ionone in FD-S was significantly higher than that in FD-A (Table 3). The second volatile with OAV > 1 was 5,6-epoxy-β-ionone, which is related to sweet, fruity, and floral aromas and can range from 670.916 to 1118.130 in spring teas.

As we know, carotenoids function as crucial aroma precursors for the production of α/β-ionones, β-citral, β-damascenone, theaspirone, and many other volatile apocarotenoids during tea manufacturing, conferring the unique flavor profiles of tea [47]. In this study, the OAVs of β-ionones, α-ionones, and 5,6-Epoxy-β-ionone in spring tea (SH-S, FD-S) were significantly higher than that in autumn tea (SH-A, FD-A) (Table 3). As an oxidized derivative of β-carotene, β-cyclocitral was detected in four samples, and the content in FD-S was significantly higher (*p ≤* 0.05) than in FD-A. This result aligns with reports indicating significantly higher concentrations of carotenoid-source volatile compounds in spring green teas than in autumn ones [14]. Research attributes this to carotenoid regulation by light and temperature, with their content peaking in spring teas [48]. In addition, the oxidative degradation of β-carotene is affected by lipoxygenases (LOXs) with seasonal activity changes, which contribute to the formation of volatile derivatives of carotenoids [49].

As a product of glycosides, the third OAV was linalool, with a numerical range of 348.887–464.531 (Table 3). Linalool has the obvious aroma of lily, sweet, grape-like, and woody, which has an important influence on the formation of excellent aroma flavor of white tea, black tea, green tea, and oolong tea [50,51,52]. In spring, the OAV of linalool in ‘Fudingdabai’ was higher than that in ‘Sanhua1951’, which was consistent with the aroma results of the sensory evaluation (Table 1). There was no significant difference in linalool between spring tea (SH-S, FD-S) and autumn tea (SH-A, FD-A). It is worth noting that (*E*)-linalool oxide was the only compound that showed significant differences in a two-by-two comparison of the four samples. (*E*)-linalool oxide was identified as a key aroma compound in teas, which mainly exhibited floral, sweet, and fruity aromas. In this study, FD-A had the highest content of (*E*)-linalool oxide, followed by SH-A, FD-S, and SH-S. This phenomenon showed that harvest season and tea cultivar may affect the formation path of (*E*)-linalool oxide. Teas harvested in autumn contained more (*E*)-linalool oxide than teas harvested in spring in both ‘Sanhua1951’ and ‘Fudingdabai’. For black tea, a study shows that the sharp increase in the content of linalool in autumn tea is related to the increase in glycosidic aroma precursors with the growth of tea leaves [53]. This may be related to differences in tea cultivars, processing techniques, and detection methods. In addition to linalool and (*E*)-linalool oxide, ocimene is also derived from geranylpyrophosphate, liberated by ocimene synthase [54]. Ocimene was deemed the key differential odorant affecting the aroma quality of the black teas produced on different dates in the spring season [36]. In this study, the fourth OAV was ocimene, possessing a herbal aroma, with a numerical range of 108.301–152.647 (Table 3), and there were no significant differences in a two-by-two comparison of the four groups.

Moreover, the volatile compounds with OAV > 10 were (*E, E*)-2,4-nonadienal, undecylic aldehyde, decanal, methyl salicylate, and 3,5-Octadien-2-one, which contributed significantly to the aroma profile and dominated the flavor of white teas. Notably, undecylic aldehyde and 3,5-octadien-2-one were identified in spring teas (SH-S, FD-S), which were not identified in autumn teas (SH-A, FD-A). Inversely, (*E, E*)-2,4-nonadienal was identified in autumn teas (SH-A, FD-A), which were not identified in spring teas (SH-S, FD-S) (Table 3). As a lipid degradation product, (E, E)-2,4-Heptadienal has contributed to the flowery attribute most and can be the key volatiles of Chinese green teas in different grades [55,56] and Pu-erh tea at different storage times [57]. In this study, there was a significant difference in the OAV of (E, E)-2,4-heptadienal between SH-S and SH-A, while no significant differences were in other samples. According to the literature reports, (*E, E*)-2,4-heptadienal was the key aroma-active compound in food samples, such as red mullet, milk, and African *Oryza glaberrima* rice [58,59,60], which the changes of content were caused by the living environment of animals and plants and processing methods. The changes in light and temperature in harvest seasons may also be the reason for the decrease in the content of (*E, E*)-2,4-heptadienal between SH-S and SH-A.

Among 30 volatile compounds, based on the principles of OAV > 1, VIP > 1, and *p* < 0.05, for ‘Sanhua1951’, 18 volatile compounds were selected as the key differential volatile compounds in white teas between spring and autumn, including (*E*, *E*)-2,4-nonadienal, (*E*, *E*)-2,4-heptadienal, (*E*)-2-decenal, 2-undecen-1-al, (*E*)-Linalool oxide, geraniol, (*Z*)-jasmone, α-ionone, β-ionone, δ-cadinene, naphthalene, 2-pentylfuran, undecylic aldehyde, α-cedrol, 3,5-octadien-2-one, 3-tridecanone, 5,6-epoxy-β-ionone, and 4-nonanolide. For ‘Fudingdabai’, 19 volatile components were selected as the key differential volatile components of white teas between spring and autumn, including Dimethyl sulfide, (*E*, *E*)-2,4-nonadienal, (*E*)-2-decenal,2-undecen-1-al, (*E*)-Linalool oxide, geraniol, (*Z*)-jasmone, α-ionone, β-ionone, β-pinene, δ-cadinene, naphthalene, undecylic aldehyde, α-cedrol, 3,5-octadien-2-one, 3-tridecanone, 5,6-epoxy-β-ionone, 4-nonanolide, and β-cyclocitral. Notably, for combined VIP > 1, *p* < 0.05, and fold change ≥2 or ≤0.5, (*E*)-linalool oxide, (*Z*)-jasmone, and δ-cadinene were identified in different seasons in two cultivars. In summary, the above-mentioned representative volatile compounds, contributing fragrance and fruity flavor characteristics, played an essential role in distinguishing the harvest seasons of two cultivars of white teas in southwestern China.

**Table 3:** Key compounds of four white teas (OAV > 1).

No.	Index	Odor Description ^a^	Threshold μg/L	OAV			
				SH-S	SH-A	FD-S	FD-A
1	Dimethyl sulfide	green, fresh	1.1 [61]	2.587 ^ab^	2.408 ^ab^	3.039 ^a^	1.850 ^b^
2	Hexanal	fresh, green, grass, fruity, apple	4.5 [62]	0.624 ^a^	1.310 ^a^	0.583 ^a^	1.292 ^a^
3	Nonanal	fresh, fruity, floral,	1 [62]	3.747 ^a^	7.05 ^a^	2.937 ^a^	4.789 ^a^
4	(E)-2-Nonenal	cucumber flavor	0.4 [62]	3.509 ^a^	3.645 ^a^	3.806 ^a^	3.613 ^a^
5	Decanal	soap, orange peel, tallow	0.1 [62]	16.894 ^a^	18.663 ^a^	16.601 ^a^	19.326 ^a^
6	(E,E)-2,4-Nonadienal	fresh, cucumber-like	0.062 [62]	0.000 ^b^	14.396 ^a^	0.000 ^b^	14.836 ^a^
7	(E,E)-2,4-Heptadienal	floral	0.03 [62]	85.000 ^a^	50.000 ^b^	62.500 ^ab^	63.130 ^ab^
8	(E)-2-Decenal	fat	0.3 [62]	5.412 ^a^	0.000 ^b^	6.732 ^a^	0.000 ^b^
9	2-Undecen-1-al	green, fresh	0.78 [63]	1.498 ^a^	0.000 ^b^	1.977 ^a^	0.000 ^b^
10	Oct-1-en-3-ol	grass, oily, earthy, fungal, vegetative-like, fresh, mushroom-like	1 [62]	0.881 ^a^	0.909 ^a^	1.199 ^a^	1.158 ^a^
11	(E)-Linalool oxide	fresh, citrus, fruity	6 [53]	0.976 ^d^	2.028 ^b^	2.014 ^c^	4.294 ^a^
12	Linalool	floral, sweet,grape-like, woody	0.22 [62]	348.887 ^b^	387.966 ^ab^	464.531 ^a^	409.690 ^a^
13	Geraniol	rose-like, sweet, waxy	40 [62]	2.491 ^a^	1.302 ^b^	2.702 ^a^	1.395 ^b^
14	(Z)-Jasmone	floral, Jasmin, woody, herbal	1.9 [62]	4.583 ^b^	1.120 ^c^	6.359 ^a^	0.804 ^c^
15	α-Ionone	sweet, fruity, woody, floral	0.4 [62]	8.494 ^a^	4.759 ^b^	11.499 ^a^	4.717 ^b^
16	β-ionone	sweet, fruity, woody, floral	0.007 [62]	2406.334 ^a^	1439.131 ^b^	3375.147 ^a^	1475.371 ^b^
17	β-Pinene	pine,turpentine	6 [62]	0.000 ^b^	0.000 ^b^	0.000 ^b^	1.054 ^a^
18	Ocimene	herbal, sweet	0.02 [62]	108.301 ^a^	128.897 ^a^	152.647 ^a^	126.525 ^a^
19	δ-Cadinene	herbal, woody	1.5 [45]	3.734 ^a^	1.125 ^b^	4.206 ^a^	1.428 ^b^
20	Methyl salicylate	mint-like	40 [62]	1.098 ^a^	1.311 ^a^	1.038 ^a^	1.153 ^a^
21	Naphthalene	pungent	0.44 [64]	4.194 ^a^	2.849 ^b^	4.997 ^a^	2.704 ^b^
22	2-Pentylfuran	floral,milk-like, beany, earthy	6 [62]	1.253 ^a^	0.000 ^b^	0.000 ^b^	0.000 ^b^
23	Undecylic aldehyde	fresh, orange peel, fat	0.014 [62]	45.072 ^a^	0.000 ^b^	59.197 ^a^	0.000 ^b^
24	α-Cedrol	woody	0.5 [63]	0.000 ^b^	3.500 ^a^	0.000 ^b^	3.312 ^a^
25	3,5-Octadien-2-one	fruity, green, grassy	0.15 [65]	60.986 ^b^	0.000 ^c^	100.4 ^a^	0.000 ^c^
26	3-Tridecanone	waxy	0.5 [63]	0.000 ^b^	1.539 ^a^	0.000 ^b^	1.423 ^a^
27	5,6-Epoxy-β-ionone	sweet, fruity, woody, floral	0.007 [66]	670.916 ^a^	0 ^b^	1118.136 ^a^	0 ^b^
28	4-Nonanolide	fruity	0.28 [62]	3.348 ^a^	0.000 ^b^	3.110 ^a^	0.000 ^b^
29	β-Cyclocitral	fresh, sweet	3 [62]	1.070 ^ab^	0.940 ^ab^	1.140 ^a^	0.750 ^b^
30	Ethyl nonylate	fruity, floral	1.2 [21]	1.210 ^a^	0.207 ^a^	1.230 ^a^	0.330 ^a^

Note: Different letters in the same line indicate a significant difference among white teas (*p* < 0.05). a: https://www.flavornet.org/flavornet.html (accessed on 15 March and 2 April 2025). Compilations of flavor threshold values in water and other media.

## 4. Conclusions

In this study, the sensory characteristics and volatile profiles of white teas harvested in spring and autumn were investigated. Through a sensory evaluation of ‘Sanhua1951’ and ‘Fudingdabai’, it was found that spring white teas (SH-S, FD-S) were superior to autumn teas (SH-A, FD-A) in appearance, aroma, and taste. Significant differences were observed in the levels of catechins, caffeine, tea polyphenols, and amino acids between the four groups. Specifically, the contents of EGC, soluble sugar, and amino acids were significantly higher in spring teas compared to autumn teas (*p* < 0.05). Conversely, the levels of ECG, C, caffeine, and total tea polyphenols were significantly lower in spring teas than in autumn teas (*p* < 0.05). These findings highlight distinct seasonal variations in the biochemical composition of teas. A total of 90 volatile compounds in white teas from two cultivars and harvest seasons were identified through HS-SPME–GC–MS analysis, which were divided into 9 chemical families. Aldehydes, alcohols, hydrocarbons, esters, and ketones were the main types of volatile compounds in white teas, among which aldehydes and alcohols were the most abundant in terms of both number and content. Spring white teas (SH-S, FD-S) contained a higher content of volatile compounds than autumn white teas (SH-A, FD-A). According to the OPLS-DA model, 52 and 57 differential volatile compounds (VIP > 1, *p* < 0.05, and fold change ≥ 2 or ≤0.5) were identified in SH-S vs. SH-A and FD-S vs. FD-A, including (*Z*)-linalool oxide, (*E*)-linalool oxide, styrene, phenylethyl alcohol, (*Z*)-citral, etc. The odor active value (OAV) results indicated that 30 key differential volatile compounds (OAV > 1) were determined in four groups, among which β-ionone, 5,6-epoxy-β-ionone, linalool, and (*E*)-linalool oxide exhibited particularly high OAVs and contributed more to pekoe and floral aroma. Moreover, 18 and 19 compounds were selected as the key volatile compounds (OAV > 1, VIP > 1, *p* < 0.05, and fold change ≥ 2 or ≤0.5) in SH-S vs. SH-A and FD-S vs. FD-A. Notably, (*E*)-linalool oxide, (*Z*)-jasmone, and δ-cadinene were identified in each cultivar, which may be characteristic volatile compounds that distinguish the harvest seasons of white teas. These findings suggest their potential as seasonal markers, paving the way for the development of the white teas ’Sanhua1951’ and ’Fudingdabai’.

## Figures and Tables

**Table 1:** Sensory assessment of ‘Sanhua1951’ and ‘Fudingdabai’ from different seasons.

Tea Cultivars	Tea Cultivars	Appearance		Aroma		Taste	
		Comment	Score	Comment	Score	Comment	Score
SH-S	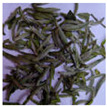	more thickset buds, fat and bold, more tippy	98 ± 1.07 ^a^	more pekoe aroma, floral, a little fresh and woody	92.94 ± 1.32 ^a^	fresh and brisk taste, sweet, mellow	92.06 ± 1.05 ^b^
FD-S	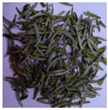	thickset buds, fat and bold, more tippy	94.87 ± 1.03 ^b^	more pekoe aroma, lasting floral, a little fresh and woody	94.81 ± 1.16 ^a^	more fresh and brisk taste, sweeter, more mellow	94.94 ± 0.90 ^a^
SH-A	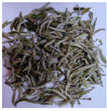	thin buds; fewer trichomes	92.94 ± 1.02 ^c^	a little pekoe, floral, and fruity	88.00 ± 1.53 ^b^	astringency, more bitterness	84.88 ± 1.03 ^c^
FD-A	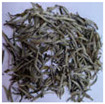	thin buds; fewer trichomes	89.63 ± 1.53 ^d^	a little pekoe, floral, and fruity	89.00 ± 1.44 ^b^	a little bitterness	86 ± 1.44 ^c^

SH-S: Sanhua1951, spring; FD-S: Fudingdabai, spring; SH-A: Sanhua1951, autumn; FD-A: Fudingdabai, autumn. Different lowercase letters in the same column indicate a significant difference among white teas (*p* < 0.05).

**Table 2:** Main biochemical components of four tea groups.

Biochemical Components (mg/g)	SH-S	FD-S	SH-A	FD-A
EC	0.26 ± 0.03 ^c^	1.66 ± 0.02 ^b^	1.61 ± 0.2 ^b^	2.85 ± 0.05 ^a^
ECG	0.66 ± 0.01 ^b^	0.3 ± 0.01 ^b^	24.37 ± 1.58 ^a^	20.44 ± 15.17 ^a^
EGC	3.76 ± 0.03 ^a^	3.9 ± 0.09 ^a^	1.67 ± 0.35 ^c^	2.47 ± 0.25 ^b^
EGCG	68.48 ± 0.49 ^b^	80.36 ± 2.66 ^a^	67.07 ± 2.1 ^b^	68.73 ± 0.64 ^b^
C	15.58 ± 0.37 ^a^	14.47 ± 0.86 ^a^	18.63 ± 0.6 ^a^	17.73 ± 0.06 ^a^
Caffeine	35.52 ± 0.5 ^c^	34.26 ± 0.35 ^c^	47.87 ± 1.6 ^a^	45.33 ± 0.58 ^b^
Free amino acid	56.23 ± 0.4 ^b^	62.37 ± 2.05 ^a^	47 ± 1.0 ^d^	41.67 ± 1.15 ^c^
Tea polyphenol	159.34 ± 4.34 ^b^	149.59 ± 5.53 ^c^	204.33 ± 3.06 ^a^	195.33 ± 1.53 ^a^
Soluble sugar	31.86 ± 1.89 ^a^	33.86 ± 1.19 ^a^	22.85 ± 1.73 ^c^	27.43 ± 1.71 ^b^
Water extracts content	475.60 ± 1.44 ^a^	476.47 ± 2.37 ^a^	470.33 ± 3.51 ^b^	471.67 ± 1.53 ^ab^

Note: Different lowercase letters in the same line indicate a significant difference among white teas (*p* < 0.05). EC, epicatechin; ECG, epicatechin gallate; EGC, epigallocatechin; EGCG, epigallocatechin-3-gallate; C, catechin.

## Data Availability

The original contributions presented in this study are included in the article/Supplementary Materials. Further inquiries can be directed to the corresponding authors.

## Supplementary Materials

The following supporting information can be downloaded at: https://www.mdpi.com/article/10.3390/foods14101795/s1, Table S1: Volatile compounds were detected by HS-SPME-GC-MS in 4 white tea samples. Table S2: Numbers of different volatile compounds of whites teas. SH-S: Sanhua1951, spring; FD-S: Fudingdabai, spring; SH-A: Sanhua1951, autumn; FD-A: Fudingdabai, autumn. Table S3: VIPs of volatile compounds between SH-S and SH-A with VIP > 1. Table S4: The key volatile compounds (VIP > 1, *p* ≤ 0.05, fold change ≥ 2 or ≤0.5) between SH-S and SH-A. Table S5: VIPs of volatile compounds between FD-S and FD-A with VIP > 1. Table S6: The key volatile compounds (VIP > 1, *p* ≤ 0.05, fold change ≥ 2 or ≤0.5) between FD-S and FD-A.

## Abbreviations

The following abbreviations are used in this manuscript:

SH-S	Sanhua1951 harvested in March
FD-S	Fudingdabai harvested in March
SH-A	Sanhua1951 harvested in August
FD-A	Fudingdabai harvested in August
PCA	Principal component analysis
OPLS-DA	orthogonal projections to latent structures–discriminant analysis
VIP	Variable importance for projection
OAV	Odor activity values
